# Cameroonian medicinal plants: a bioactivity versus ethnobotanical survey and chemotaxonomic classification

**DOI:** 10.1186/1472-6882-13-147

**Published:** 2013-06-26

**Authors:** Fidele Ntie-Kang, Lydia Likowo Lifongo, Luc Meva’a Mbaze, Nnange Ekwelle, Luc C Owono Owono, Eugene Megnassan, Philip N Judson, Wolfgang Sippl, Simon M N Efange

**Affiliations:** 1 Chemical and Bioactivity Information Centre, Department of Chemistry, Faculty of Science, University of Buea, P. O. Box 63 Buea, Cameroon; 2 CEPAMOQ, Faculty of Science, University of Douala, P.O. Box 8580 Douala, Cameroon; 3 Department of Chemistry, Faculty of Science, University of Douala, P. O. Box 24157, Douala, Cameroon; 4 Klinikum Südstadt, Südring 81, 18055 Rostock, Germany; 5 Laboratory for Simulations and Biomolecular Physics, Ecole Normale Supérieure, University of Yaoundé I, P.O. Box 47, Yaoundé, Cameroon; 6 Laboratory of Fundamental and Applied Physics, University of Abobo-Adjame, Abidjan 02 BP 801 Cote d’Ivoire; 7 Chemical and Bioactivity Information Centre, 22-23 Blenheim Terrace, Woodhouse Lane, Leeds LS2 9HD UK; 8 Department of Pharmaceutical Sciences, Martin-Luther University of Halle-Wittemberg, Wolfgang-Langenbeck Str. 4, 06120 Halle(Saale), Germany

**Keywords:** Biological activities, Chemotaxonomy, Ethnobotany, Medicinal plants, Natural products

## Abstract

**Background:**

In Cameroon herbs are traditionally used to meet health care needs and plans are on the way to integrate traditional medicine in the health care system, even though the plans have not been put into action yet. The country however has a rich biodiversity, with ~8,620 plant species, some of which are commonly used in the treatment of several microbial infections and a range of diseases (malaria, trypanosomiasis, leishmaniasis, diabetes and tuberculosis).

**Methods:**

Our survey consisted in collecting published data from the literature sources, mainly from PhD theses in Cameroonian university libraries and also using the author queries in major natural product and medicinal chemistry journals. The collected data includes plant sources, uses of plant material in traditional medicine, plant families, region of collection of plant material, isolated metabolites and type (e.g. flavonoid, terpenoid, etc.), measured biological activities of isolated compounds, and any comments on significance of isolated metabolites on the chemotaxonomic classification of the plant species. This data was compiled on a excel sheet and analysed.

**Results:**

In this study, a literature survey led to the collection of data on 2,700 secondary metabolites, which have been previously isolated or derived from Cameroonian medicinal plants. This represents distinct phytochemicals derived from 312 plant species belonging to 67 plant families. The plant species are investigated in terms of chemical composition with respect to the various plant families. A correlation between the known biological activities of isolated compounds and the ethnobotanical uses of the plants is also attempted. Insight into future direction for natural product search within the Cameroonian forest and Savanna is provided.

**Conclusions:**

It can be verified that a phytochemical search of active secondary metabolites, which is inspired by knowledge from the ethnobotanical uses of medicinal plants could be very vital in a drug discovery program from plant-derived bioactive compounds.

## Background

The African continent holds an enormous resource in terms of floral biodiversity and its medicinal plants have remained a main reservoir of phytochemicals for pharmaceutical drug development [1,2]. The above argument could be strengthened by the fact that the local populations, especially South of the Sahara, have depended on medicinal plants as the main source of remedy for the treatment of several medical disorders over the past centuries [3]. The World Health Organization (WHO) defines traditional medicine as practices, knowledge and belief systems which use minerals, plants and animal based remedies, spiritual therapies and exercises to prevent, treat and maintain well being [4]. According to WHO, 80% of the world’s population used natural remedies and traditional medicines [4,5]. This is particularly the case in Africa, as well as in most developing countries, where a high proportion of the population still resorts to traditional medicine for their primary health care. Despite the advances in Western medicine (WM), African traditional medicine (ATM) has gained renewed interest in the health care services throughout the continent. This has probably been motivated by the rapidly increasing awareness of the potential and curative abilities of alternative medicines, especially from the use of medicinal plants, as well as the inadequate access to WM and physicians and the high cost for Western drugs [6]. ATM utilises medicinal plants which are traditionally taken as concoctions and infusions [7]. The argument for the local African populations resorting to traditional remedies could also be partly justified by the fact that natural product inspired molecules represented 80% of drugs that had been put into the drug market by 1990 [8-10]. The above figures have been the motivating factor behind the efforts of several research teams spread across the African continent, which have been actively engaged in an ethnobotanical/bioassay-guided search for active principles from medicinal plants which have been employed in ATM. The aim has been to extract, isolate and structurally characterise plant metabolites which could be developed into modern therapeutic agents. This process has often been coupled with biological screening of the isolated compounds, with a view to validating the uses of the plants in traditional medicine, thus identifying the molecular structures that could be further developed into drugs. Very often, success in this endeavour could be attributed to the closeness of the collaboration between experts in various fields like traditional healers, ethnobotanists, plant taxonomists, phytochemists, and biochemical screeners [11]. Since ATM often employs the preparation of macerations, preparation of decoctions of stem barks, leaves and roots, burning of leaves and using the derived ashes, etc., a rather straightforward procedure could be to test for the activity of the crude extracts of these plant parts. Such research has been reported and the results for activity as well as the toxicity of crude extracts serve as a guide towards the development of “total” extracts which are often directly conditioned and used in the low cost “modern” traditional treatments, affordable to the local populations [12]. Such approaches could however present serious drawbacks when situated within a modern context of WM. In WM, modern pharmaceutical companies have elaborated expensive Research and Development (R&D) procedures that work with highly sophisticated solid support combinatorial synthesis followed by highthroughput screening (HTS), *in vivo* animal model evaluations and clinical trials in order to determine appropriate doses, modes of administration and possible side effects before marketing the finished products (drugs). It could however be noted that one of the strongest arguments why ATM has become the main stay in the health care system of the continent, despite the above mentioned drawbacks, is that its local populations are often incapable of purchasing the expensive Western drugs [13].

**Figure 1:**
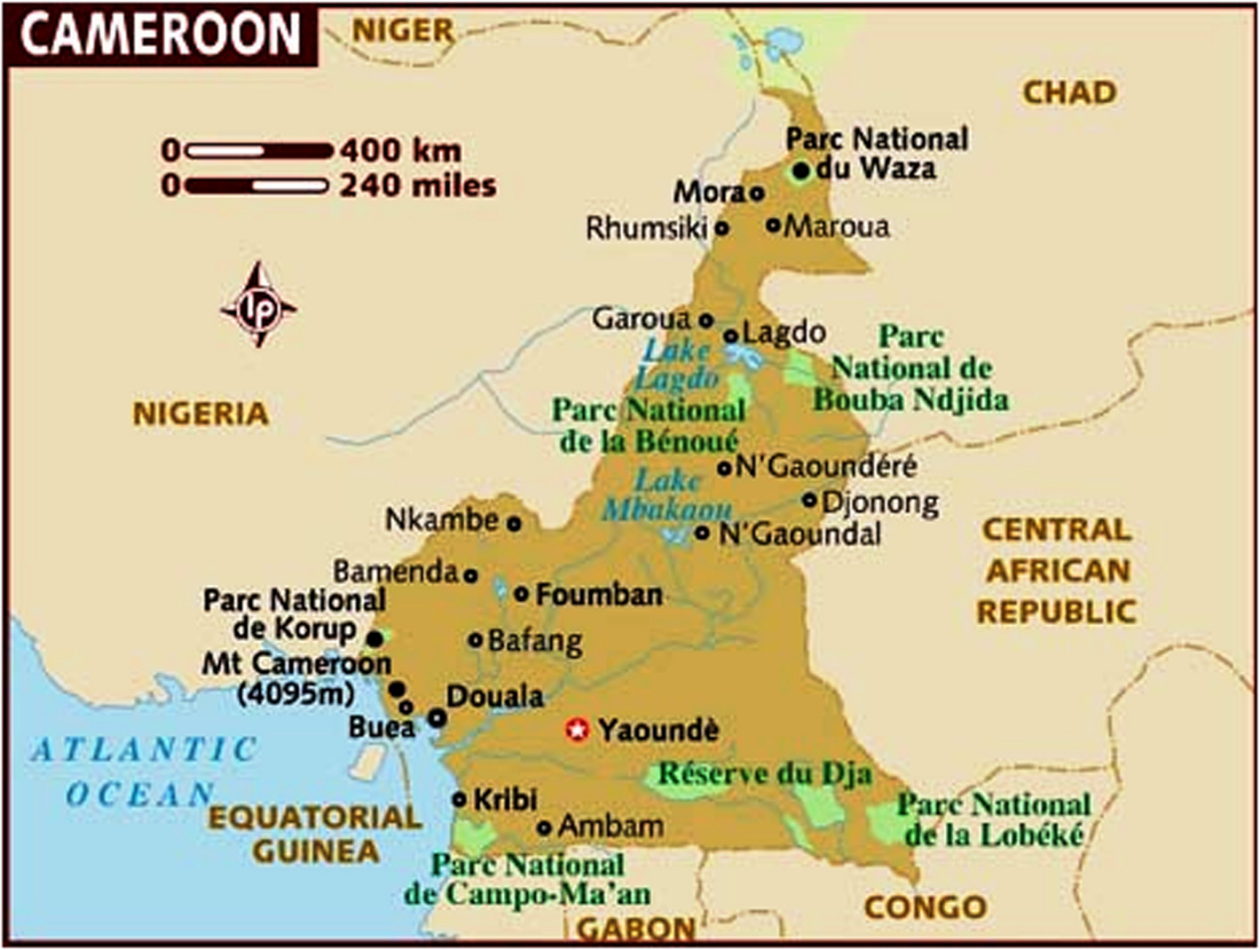
Cameroon (in brown) and its surrounding neighbours.

In Cameroon, located in the core of the continent, and bounded by Nigeria (to the West), Chad (to the North and North East), Central Africa Republic to the South East and Gabon, Congo and Equatorial Guinea to the South (Figure 1), the picture is not very different from what is seen throughout the rest of the continent. The mortality patterns reflect high levels of infectious diseases and the risk of death during pregnancy and childbirth, in addition to cancers, cardiovascular diseases and chronic respiratory diseases which account for most deaths in the developed countries [14]. It has been reported that only 3 out of 20 patients are able to buy prescribed drugs in hospitals and only 1 out of every 1000 patients is able to consult a specialist [15]. As a result there is a rich tradition in the use of herbal medicines for the treatment of several ailments and plans are on the way to integrate traditional medicine in the health care system, even though the plans have not been put into action yet [16]. Cameroon however has a rich biodiversity, with ~8,620 plant species [17,18], some of which are commonly used in the treatment of several microbial infections [19] and a range of neglected tropical diseases, including malaria, trypanosomiasis, leishmaniasis, diabetes, tuberculosis, etc. [15]. Adjanohoun *et al*. have provided a useful review on a collection of 414 plants used in traditional medicine in Cameroon, belonging to 95 plant families [20]. However, in addition to the fact that the formulations of the plant materials to be used as drugs in ATM have not been validated in a well documented and universally accepted pharmacopoeia, no details are given regarding the chemical composition of these plants. Jiofack *et al*. added to this by providing the formulation for 289 plant species belonging to 89 families, which have been used in two ethnoecological regions (Littoral-South West, and Sudano-Sahelian regions) of Cameroon for the treatment of various ailments, including amoebiasis, boils, cough, dermatitis, diarrhoea, dysentery, fever, gastritis, gonorrhoea, malaria, male sexual disorders, ovarian cysts, rheumatism, sexually transmitted diseases, sterility, syphilis, typhoid, and wounds [21-23]. Jiofack *et al*. also added a list of 140 plant species belonging to 60 families, which are used to treat a wide range of ailments in the upper Nyong valley forest in Cameroon [24]. Simbo also carried out an ethnobotanical survey around Babungo in the North West region of Cameroon, in which 107 plant species from 54 plant families (mostly from the Asteraceae family), used for the treatment of 55 health problems, were identified [25], while Focho *et al*. identified 82 species of trees belonging to 70 genera and 42 families used by the local people in traditional medicine around Fundong in the North West region to treat 48 human ailments [26]. Ngono *et al*. carried out a survey in which plants used in the treatment of viral diseases in the Centre and South regions were identified [7]. Noumi *et al*. identified 26 plant species used to treat hypertension in the Bafia area of the Centre region [27], 22 medicinal plant species used in the treatment of urinary lithiasis in the Littoral region [28], 29 plants from 24 families used in the treatment of asthma in the Nkongsamba region [29], 29 medicinal plants used in the treatment of peptic ulcers around Bagangte in the West region [30], as well as 50 plants from 33 families which are used in the treatment of syphilis in Ebolowa in the South region, Cameroon [31]. Mpondo *et al*. conducted an ethnobotanical survey among the Douala communities and identified more than 100 genera which have been used in the treatment of diverse diseases like stomach ache, tooth ache, diabetes, cough, yellow fever, amoebic dysentery, anaemia, intestinal worms, fever and diarrhoea [32-38]. Ngo Bum *et al*. also reported 23 plants species used in Cameroon and Central Africa to treat epilepsy [39], while Betti identified 102 plants from 97 genera and 51 families used mainly to treat cough, lactation failure, malaria, wounds, and toothache among the Baka Pygmies in the Dja biosphere reserve [40]. Sandberg *et al*. have also reported a collection of 32 botanically identified medicinal plants from the Mount Cameroon slope, made by Swedish settlers at the beginning of the last century, which has been donated to the Karolinska Institutet, Sweden [41]. In all the above cases, no details were given about the active principles contained in these plants, which render them useful in ATM. Titanji *et al*. have however previously presented a review of about 217 cited species used in the treatment of malaria in Cameroon, among which about 100 potential leads for the development of new antimalarials had been isolated from 26 species [42].

In a recent paper, we have reported the results of a survey of the chemical composition of 234 plant species (representing 55 families) from the Cameroonian rainforest, from which 1,859 distinct compounds had been isolated or derived [43]. Our focus had been to highlight the medicinal value and virtues of the isolated phytochemicals by discussing the various measured biological activities and evaluating the “drug-like” properties by use of Lipinski’s “Rule of Five” [44]. In the current paper, we have enriched the data collection to include phytochemicals from 312 distinct plant species from 67 families, for which biological activities of 46.3% of the 2,769 pure compounds have been determined, and among which 32.7% of the compounds were identified or derived from Cameroonian medicinal plants for the very first time. In addition, we present and discuss 98 compounds whose measured biological activities correlated well with the ethnobotanical uses of the plants in ATM. An attempt to relate the chemical composition of the plant species to the families and genera is also carried out. From our study, it can be verified that a phytochemical search of active secondary metabolites, which is inspired by knowledge from the ethnobotanical uses of medicinal plants could be very vital in a drug discovery program from plant-derived bioactive compounds.

## Methods

### Data sources and collection methods

The plant sources, geographical collection sites, chemical structures of pure compounds as well as their spectroscopic data, were retrieved from literature sources comprising of data collected from 30 PhD theses, 556 articles from 75 peer reviewed journals, 7 unpublished conference presentations (from personal communication with the authors) and 2 textbook chapters, spanning the period 1971 to 2013. A full list of journals consulted is given in the supplementary material (Additional file 1). Our survey consisted in collecting published data from the literature sources, mainly from PhD theses in Cameroonian university libraries and also using the author queries in major natural product and medicinal chemistry journals. The collected data includes plant sources, uses of plant material in traditional medicine, plant families, region of collection of plant material, isolated metabolites and type (e.g. flavonoid, terpenoid, etc.), measured biological activities of isolated compounds, and any comments on significance of isolated metabolites on the chemotaxonomic classification of the plant species (as commented in the literature). This data was compiled on a excel sheet and analysed.

## Results and discussion

### Chemotaxonomy of Cameroonian medicinal plants

Chemotaxonomy or chemosystematics is the attempt to classify and identify organisms (originally plants), according to demonstrable differences and similarities in their biochemical compositions. Generally speaking, it has been observed that plants of the same family usually synthesize compounds of similar classes due to the presence of similar classes of enzymes and hence similar biosynthetic pathways. Moreover, the medicinal plants surveyed are a complex of species from different groups (genera or families). This paper is not intended to give an accurate description of the chemotaxonomy of Cameroonian medicinal plants. The similarities or differences of chemical components from different medicinal plants presented herein are solely based on data published so far, by presenting trends towards the full description of the taxonomy of the studied families and species.

**Figure 2:**
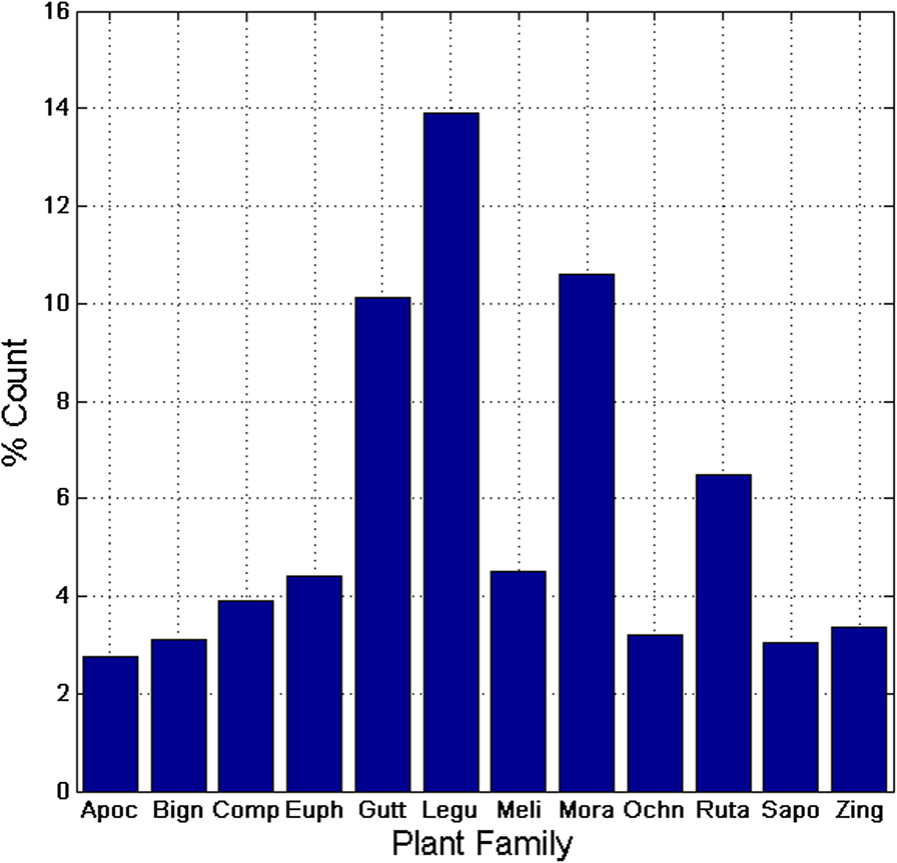
**Bar chart showing the distribution of isolated compounds by plant family.** Family names are indicated by the first four letters, e.g., Apoc = Apocynaceae.

**Figure 3:**
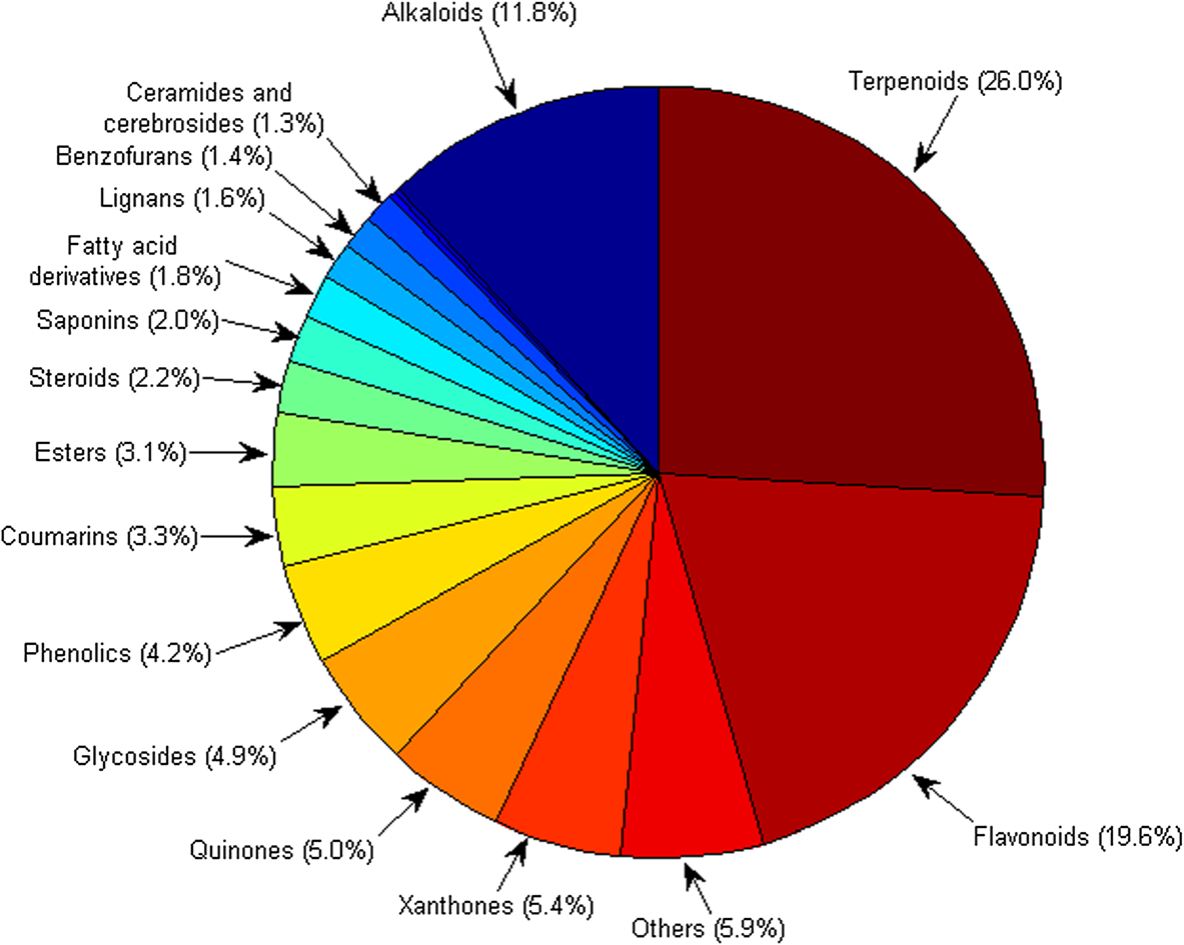
Pie chart showing the distribution by compound types.

Our initial collection was composed of 3,742 phytochemicals previously isolated from 67 families, along with 319 of some of their hemisynthetic products, giving a total of 4061 chemical structures. Removal of duplicates gave 2,770 pure compounds. In our analyses, emphasis was laid on those plant families from which at least 2.5% of the secondary metabolites have been isolated. These include, by order of merit, Leguminosae (13.9%), Moraceae (10.6%), Guttiferae (10.1%), Rutaceae (6.5%), Meliaceae (4.5%), Euphorbiaceae (4.4%), Compositae (3.9%), Zingiberaceae (3.4%), Ochnaceae (3.2%), Bignioniaceae (3.1%), Sapotaceae (3.1%) and Apocynaceae (2.8%), Figure 2. An overall distribution by compound type (based solely on unique compounds, not compound concentrations in the plants) is shown in Figure 3. This revealed that terpenoids were most abundant in Cameroonian medicinal plants (constituting 26.0% of the isolated compounds). This was followed by flavonoids (19.6%), alkaloids (11.8%), xanthones (5.4%), quinones (5.0%) and glycosides (4.9%), showing a similar trend with our previous analysis of 1,859 metabolites [43].

**Figure 4:**
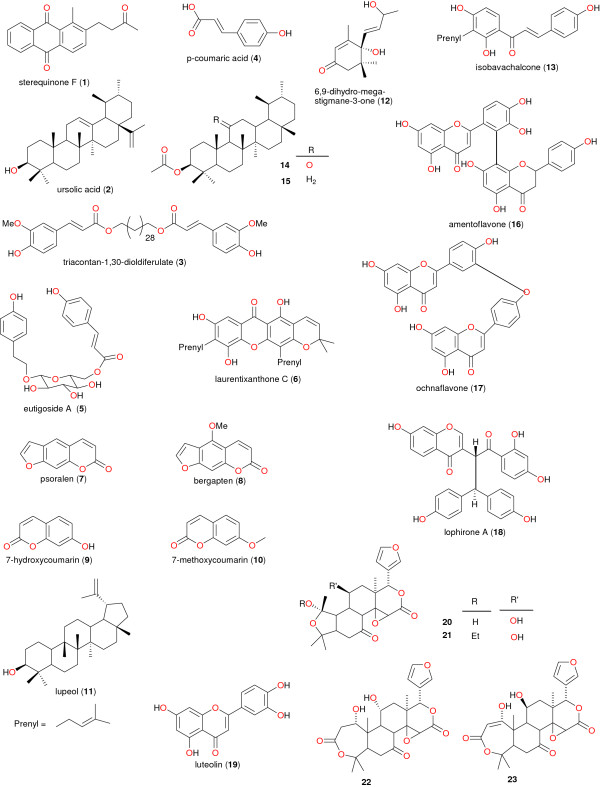
Chemical structures of taxonomic markers.

A more detailed analysis of compounds isolated per family is given in Table 1. From the Leguminosae family, it was shown that most of the isolated metabolites were flavonoids (68.3%). A similar trend was seen in the Ochnaceae and Moraceae families with respective flavonoid % compositions of 69.2% and 39.7%. The terpenoid rich families are the Meliaceae (74.4%), the Euphorbiaceae (68.5%), the Sapotaceae (43.2%) and the Zingiberaceae (70.1%), while the alkaloid rich families are the Apocynaceae (54.4%) and the Rutaceae (54.6%). From our analyses it was shown that, as in our previous paper, most of the isolated metabolites from the Guttiferae family were xanthones and quinines [43]. Moreover, within the Leguminosae family, isoflavonoids and diterpenoids, with insecticidal, piscicidal, and molluscicidal properties, are taxonomic markers of the genus *Millettia*[45]. Meanwhile within the Bignoniaceae family, sterequinone F (1), ursolic acid (2), triacontan-1,30-dioldiferulate (3) and *p*-coumaric acid (4) have been identified as the taxonomic markers for the genus *Stereospermum*, while the glycoside eutigoside A (5) has been used to differentiate *S*. *accuminatissimum* from other members of the genus [46-48]. The chemical structures of the taxonomic markers are shown in Figure 4. Within the Guttiferae family, laurentixanthone C (6) was identified to be the chemotaxonomic marker of the genus *Vismia*[49]. As for the Moraceae family, the coumarins psoralen, bergapten, 7-hydroxycoumarin and 7-methoxycoumarin (7 – 10 respectively) and lupeol (11) have been identified as taxonomic markers for the *Dorstenia* genus [50]. It was however generally observed that species of the genus *Dorstenia* harvested from across the African continent are particularly unique in that they produce a wide range of chalcones and bichalcones, prenylated flavonols, benzofuran derivatives, and furocoumarins [51]. It was also noticed that 6,9-dihydro-megastigmane-3-one (12) has been isolated from the genus *Treculia* (both *T*. *africana* and *T*. *Acuminata*). This compound may tentatively be considered as a marker of the *Treculia* genus. Isobavachalcone (13), previously isolated from several species of the genus *Dorstenia*, can be used to establish intertribal relationship between the two genera *Treculia* and *Dorstenia*[52]. Moreover, the isolation of compounds 8, 14 and 15 from *Dorstenia* and *Ficus* species indicate that these compounds could be chemotaxonomic markers for the *Dorstenia* genus and confirms that the genera *Dorstenia* and *Ficus* are closely related taxonomically [53]. Regarding the Ochnaceae, the biflavonoid amentoflavone (16) could be as well regarded as the taxonomic marker of the genus *Ouratea*, ochnaflavone (17) used to identify species of both *Ouratea* and *Ochna*, while lophirone A (18) could be associated with almost all genera in the family [54,55]. Meanwhile, the presence of the flavonoid luteolin (19) in *Lophira alata* could be used to distinguish between the two species *L*. *alata* and *L*. *Lanceolata*[56]. Among species of the Rutaceae family, the accumulation of acridone alkaloids displays a common biogenetic trend in the genus *Citrus*, and could be used to distinguish this genus from the counterpart *Afraegle*[57]. Meanwhile, within this same family, the unique presence of the tetranortriterpenoids (20–23) within the genus *Clausena*, could reveal that these compounds are of taxonomic interest [58]. Within the Zingiberaceae family labdane diterpenes may represent a chemotaxonomic marker of the genus *Aframomum*. However, *A*. *arundinaceum* is one of the few species of *Aframomum* from which sesquiterpenoids are reported [59,60].

**Table 1:** Summary of the chemical composition of the remarkable plant families with abundant phytochemicals isolated

**Plant family**	**% of isolated compounds**	**Remarkable compound classes (% composition)**	**Genera studied**
Apocynaceae	2.8	Alkaloids (54.4%)	*Holarrhena*, *Picralima*, *Tabernathe*, *Voacanga*, *Picralima*, *Rauvolfia* and *Plumeria*
Bignoniaceae	3.1	Quinones (26.3%)	*Kigelia*, *Newbouldia*, *Spathodea* and *Stereospermum*
Compositae	3.9	Terpenoids (54.1%)	*Chromoleana*, *Crassocephalum*, *Crepis*, *Echinops*, *Elephantopus*, *Eupatroium*, *Helichrysum*, *Microglossa*, *Senecio*, *Tithonia* and *Vernonia*
Euphorbiaceae	4.4	Terpenoids (68.5%)	*Alchornea*, *Antidesma*, *Croton*, *Discoglypremna*, *Drypetes*, *Fontainea*, *Macaranga*, *Maesobotrya*, *Neoboutonia*, *Thecacoris* and *Uapaca*
Guttiferae	10.1	Xanthones (37.5%), Quinones (26.3%)	*Allanblackia*, *Calophyllum*, *Endodesmia*, *Garcinia*, *Harungana*, *Hypericum*, *Pentadesma*, *Psorospermum*, *Symphonia* and *Vismia*
Leguminosae	13.9	Flavonoids (68.3%)	*Cassia*, *Crotalaria*, *Eriosema*, *Erythrina*, *Guibourtia*, *Millettia*, *Tephrosia* and *Piptadenia*
Meliaceae	4.5	Terpenoids (74.4%)	*Carapa*, *Entandrophragma*, *Leplaea*, *Pterorhachis*, *Synsepalum*, *Trichilia* and *Turraeanthus*
Moraceae	10.6	Flavonoids (39.7%)	*Antiaris*, *Artocarpus*, *Dorstenia*, *Ficus*, *Melicia*, *Milicia*, *Morus*, *Treculia* and *Trilepisium*
Ochnaceae	3.2	Flavonoids (69.2%)	*Campylospermum*, *Lophira*, *Ochna* and *Ouratea*
Rutaceae	6.5	Alkaloids (54.6%)	*Afraegle*, *Araliopsis*, *Basalmocitrus*, *Citropsis*, *Clausena*, *Fagara*, *Oricia*, *Oriciopsis*, *Teclea*, *Vepris*, and *Zanthoxylum*
Sapotaceae	3.1	Terpenoids (43.2%)	*Butyrospermum*, *Chrysophyllum*, *Donella*, *Gambeya* and *Mimusops*
Zingiberaceae	3.4	Terpenoids (70.1%)	*Aframomum* and *Renealmia*

### Bioactivity versus ethnobotanical uses

**Figure 5:**
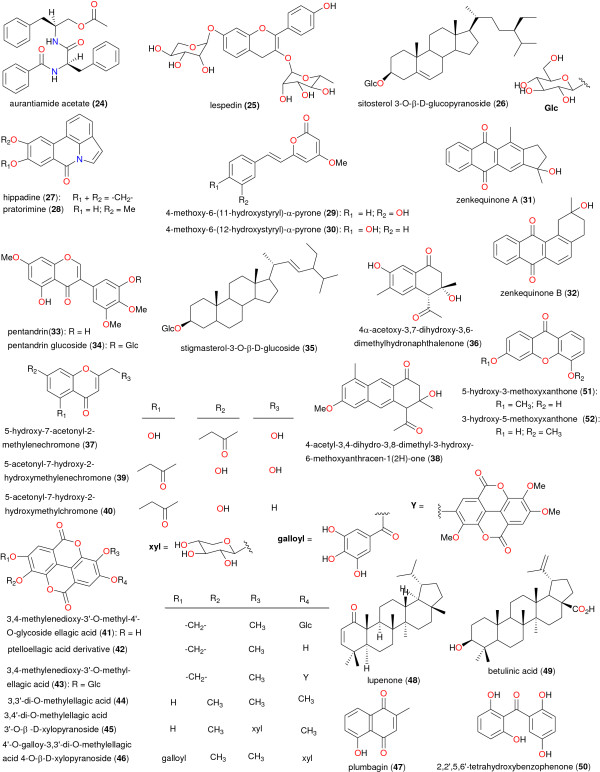
**Chemical structures of bioactive metabolites I.** Compounds 24 to 52.

**Figure 6:**
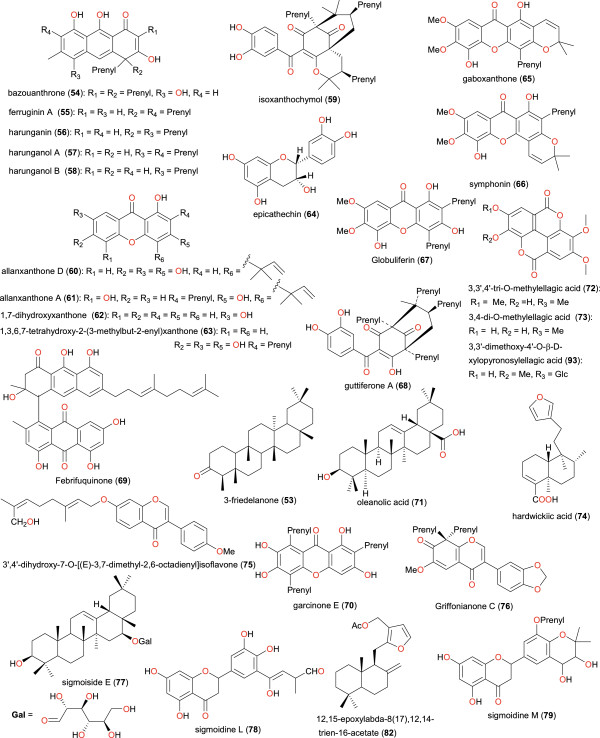
**Chemical structures of bioactive metabolites II.** Compounds 53 to 79, 82 and 93.

**Figure 7:**
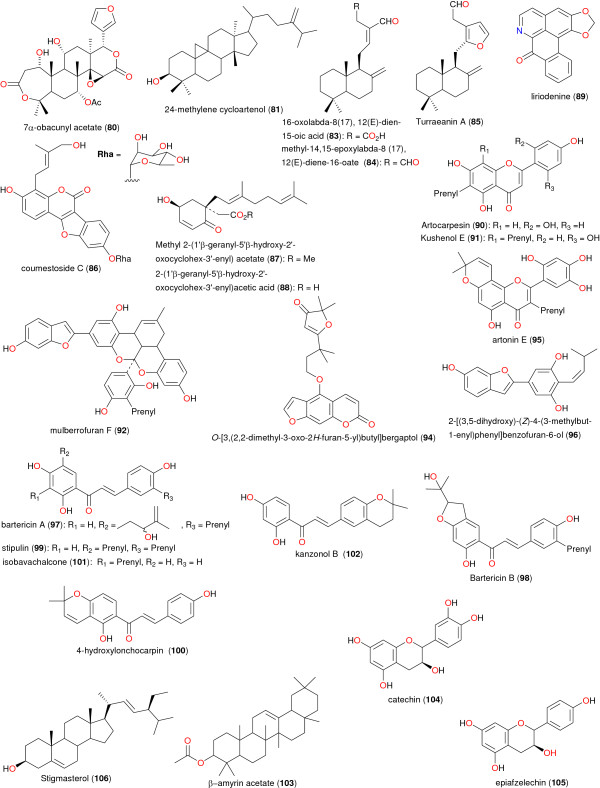
**Chemical structures of bioactive metabolites III.** Compounds 80, 81, and 83 to 106.

**Figure 8:**
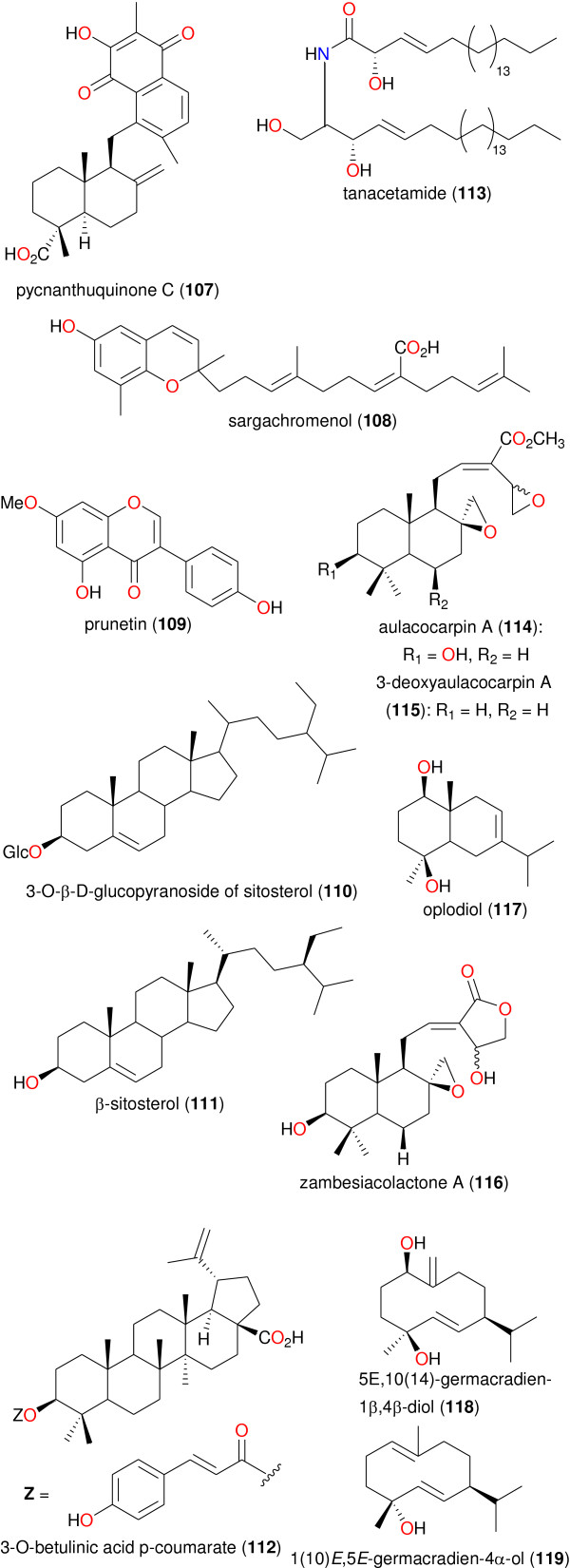
**Chemical structures of bioactive metabolites IV.** Compounds 107 to 119.

Ethnobotany is the study of how modern and indigenous societies view and use plants [61]. As previously mentioned, medicinal plants from Cameroon have been used to treat a number of ailments. In this study, our focus is on those plants from which bioactive secondary metabolites have been isolated, which validate their ethnobotanical uses. Table 2 shows a summary of the results of biological activities of the isolated compounds, which correlate with the ethnobotanical uses of the plants (while the chemical structures of the bioactive metabolites are shown in Figures 5, 6, 7 and 8). In each case, the significant uses and corresponding bioactivities are in bold characters. The activities of the selected metabolites are anti-malarial, estrogenic, antisalmonellal, hepatoprotective, antifungal, antioxidant, antidermatophytic, vasorelaxant, anticancer, antileishmanial, antimicrobial and α-glucosidase inhibition. The antimalarials with significant antiparasitic activity, which have been isolated from plants used in anti-malarial or anti-fever preparations include: betulinic acid (49), 2,2’,5,6’-tetrahydroxybenzophenone (50), 5-hydroxy-3-methoxyxanthone (51) and 3-hydroxy-5-methoxyxanthone (52) [62]; bazouanthrone (54), ferruginin A (55), harunganin (56), harunganol A (57) and harunganol B (58) [63]; isoxanthochymol (59), which exhibited an anti-malarial activity against the NF54 strain with a 50% inhibitory concentration (IC_50_) of 2.21 μM [64]; gaboxanthone (65), symphonin (66), globuliferin (67) and guttiferone A (68) [65]; garcinone E (70), with an IC_50_ of 0.20 μM, which was isolated from *Pentadesma butyracea*, concurrently with other potent anti-malarials pentadexanthone, cratoxylone and α-mangostin [66]; the limonoid 7α-obacunyl acetate (80) [67]; the homogentisic acid derivatives methyl 2-(1’β-geranyl-5’β-hydroxy-2’-oxocyclohex-3’-enyl) acetate (87) and 2-(1’β-geranyl-5’β-hydroxy-2’-oxocyclohex-3’-enyl)acetic acid (88) and the alkaloid liriodenine (89) isolated from *Glossocalyx brevipes*[66]; the flavonoids artocarpesin (90), Kushenol E (91), and the arylbenzofuran derivative mulberrofuran F (92) isolated from *Morus mesozygia*[68]; the chalcones bartericin A (97) and 4-hydroxylonchocarpin (100) [69]; 3-*O*-betulinic acid *p*-coumarate (112) isolated from *Baillonella toxisperma*, with an IC_50_ of 1.65 μM [70]; the labdane 3-deoxyaulacocarpin A (115) from *Aframomum zambesiacum*[71]; and the sesquiterpenoids oplodiol (118), 5*E*,10(14)-germacradien-1β,4β-diol (119) and 1(10)*E*,5*E*-germacradien-4α-ol (120) with respective IC_50_ values of 4.17, 1.63 and 1.54 μM [72]. In all cases, antiplasmodial activity was measured by inhibition of the chloroquine-resistant W2 *P*. *falciparum* strain with IC_50_ < 5 μM.

**Table 2:** Summary of ethnobotanical uses versus measured biological activities of isolated secondary metabolites

**Plant family**	**Plant species**	**Ethnobotanical uses**	**Part of plant studied**	**Isolated metabolites**	**Measured activity of Isolated metabolite**
Acanthaceae	*Brillantaisia lamium*	The aerial part of this plant is used in the treatment of various **microbial infections**, such as **skin diseases and infections of the urinary tract**.	aerial parts	11, 24, 25 and 26	**Antimicrobial**, Tamokou *et al*. [73]
Amaryllidaceae	*Crinum purpurascens*	The macerated leaves are used as anti-poison or as antidote of mystical poisoning. Also, an infusion of the leaves is used for the treatment of some **microbial infections**.	leaves	27 and 28	Anti-salmonellal and **antibacterial**, Nkanwen *et al*. [74]
Asteraceae	*Senecio mannii*	The plant is used to treat microbial and **fungal infections**.	aerial parts	29 and 30	**Antifungal**, Ndom *et al*. [75]
Bignoniaceae	*Stereospermum zenkeri*	The bark is to treat bronchitis; its roots and leaves are used to cure fever and **microbial infections**.	stem bark	31 and 32	**Antimicrobial**, Lenta *et al*. [48]
Bombacaceae	*Ceiba pentandra*	The plant is well reputed in for the treatment of many illnesses, such as, headache, dizziness, constipation, **skin diseases**, mental troubles, and fever.	stem bark	33 and 34	**Antifungal**, Ngounou *et al*. [76]
Caesalpiniaceae	*Cassia petersiana*	The leaves are generally used for the treatment of **typhoid fever**.	leaves	35, 36, 37, 38, 39 and 40	**Antisalmonellal activity**, Djemgou *et al*. [77], Gatsing *et al*. [78]
Combretaceae	*Pteleopsis hylodendron*	Treatment of STDs, **female sterility**, kidney and liver disorders and dropsy.	bark	41, 42, 43 and 44	Active against ***Staphylococcus aureus*,*S*. *pyogenes*, and*Baccilus cereus***, Rahman *et al*. [79]
	*Terminalia superba*	Used to treat gastroenteritis, **diabetes**, female infertility and abdominal pain.	bark	45 and 46	**α-Glycosidase inhibition**, immunoinhibitory activity, Tabopda *et al*. [80]
Ebenaceae	*Diospyros canaliculata*	Used in the treatment of whooping cough, leprosy, snake bites, scabies, skin eruptions, dysentery, eye infections, menstrual troubles, abdominal pains, wounds, ulcers, chest pains and **skin infections**.	stem bark	11, 47 and 48	**Antifungal activity**, Dzoyem *et al*. [81]
Hypericaceae, Clusiceae or Guttiferae	*Hypericum lanceolatum*	Used to treat several ailments including **malaria**, skin infections, venereal diseases, gastrointestinal disorders, tumours, and infertility, epilepsies and nerves problems.	stem bark	49, 50, 51 and 52	**Anti-malarial**, Zofou *et al*. [62]
	*Harungana madagascariensis*	Effective in the treatment of jaundice, diarrhoea, dysentery, typhoid fever, and constipation. Decoction of leaves is also used in liver problems and against anaemia. The roots and bark are used to treat **malaria**.	stem bark	49, 53, 54, 55, 56, 57 and 58	**Antiplasmodial**, Lenta *et al*. [63]
Hypericaceae, Clusiceae or Guttiferae	*Garcinia Polyantha*	The plant has some **anti-malarial property**. The yellow resinous sap (latex) is used to make a dressing for wounds.	root bark	59	This compound shows **anti-malarial activity** by strong chemosuppression of parasitic growth, Lannang *et al*. [64]
	*Allanblackia gabonensis*	Used against infections like **dysentery**, cold, and toothache.	stem bark	60, 61, 62, 63 and 64	Activity against ***Leishmania amazonensis*and antimicrobial activities** against a range of Gram-negative and Gram-positive bacteria, Azebaze *et al*. [82]
	*Symphonia globulifera*	Used to cure several diseases such as stomach and skin aches. It is also used as laxative for pregnant women and as a **general tonic**. The bark is used by traditional healers to treat **malaria**.	seed shells	65, 66, 67 and 68	**Anti-malarial**, **antioxidant**, Ngouela *et al*. [65]
	*Psorospermum febrifugum*	Plants of this genus are largely used in the African folk medicine as febrifugal, antidote against poison and purgative. They are also used as a remedy for the treatment of leprosy, **skin diseases** (such as dermatitis, scabies and eczemas) and subcutaneous wounds.	roots	69	Antimicrobial (bacteria and **fungi**), Tsaffack *et al*. [83]
	*Pentadesma butyracea*	Different parts of the plant are used in tropical African medicine to treat **fever**, coughs, constipation, bronchitis, and venereal diseases and viral infections.	fruit pericarp	70	Erythrocyte susceptibility, **antiplasmodial**, Lenta *et al*. [84]
Ixonanthaceae	*Irvingia gabonensis*	The stem bark decoction is used in the treatment of **gonorrhoea**, gastrointestinal or **hepatic disorders**, as a purgative, as well as a host of ailments. The decoction of the root barks is also used to treat diarrhoea and as mouth bath in the dental neuralgias.	stem bark	49, 53, 71, 72, 73 and 74	Antimicrobial. Compound 74 is particularly active **against*Neisseria gonorrhoeae***, confirming the ethnobotanical use of the plant in the treatment of the disease caused by this agent, Kuete *et al*. [85]
			stem bark	53	
					**Hepatoprotective activity**. This crude extract and isolated compound 53 might be useful for the prevention of toxic-induced and free radical-mediated **liver diseases**, since it has been suggested that compounds may be used as prophylactic agents, Donfack *et al*. [86]
Leguminosae-Papilionoideae	*Millettia griffoniana*	Crude extracts from root and stem bark are used to treat boils, insect bites, inflammatory affections like pneumonia and asthma, **sterility**, **amenorrhea and menopausal disorders**.	root bark	75 and 76	**Estrogenic activity**, Wanda *et al*. [45]
	*Erythrina sigmoidea*	Widely used in Cameroon to treat **syphilis**, wounds of ulcers and **female sterility**.	stem bark	77, 78 and 79	Antibacterial activity against ***Staphylococcus aureus***, Kouam *et al*. [87-89]
Meliaceae	*Entandrophragma angolense*	Used as an **anti-malarial** or antipyretic in traditional medicine.	stem bark	80 and 81	**Antiplasmodial**, Bickii *et al*. [67]
	*Turraeanthus africanus*	Treatment of **typhoid fever**.	seeds	82	**Antisalmonellal** activity against *Salmonella typhi*, *S*. *paratyphi A* and *S*. *paratyphi B*, Djemgou *et al*. [90]
		The species of this genus have been used for the treatment of cardiovascular disease, stomach ache, rheumatism pains, and asthma. The stem bark is used in the treatment of intestinal worms.	seeds	82	Antimicrobial, Djemgou *et al*. [90]
		The trunk bark and seeds of this plant are boiled together with *Carica papaya* leaves, the seeds of *Aframomum melegueta* and lime and used for treatment of **malaria and other fevers**.	seeds	83, 84 and 85	**Antiplasmodial activity**, Ngemenya *et al*. [91]
Leguminosae- Mimosoideae	*Cylicodiscus gabunensis*	Used to prepare remedies for **infectious diseases** and is known for its **antibacterial** and antiplasmodial activities.	stem bark	86	Exhibited **antimicrobial** activity against *Proteus vulgaris*, Nchancho *et al*. [92]
	*Albizia adianthifolia*	Used traditionally to treat several ailments, including **infectious and associated diseases**.	stem bark	24	Antioxidant and **antimicrobial activities**, Tamokou *et al*. [93]
Monimiaceae	*Glossocalyx brevipes*	The macerated leaves are added to **anti-fever** preparations.	leaves	87, 88 and 89	**Anti-malarial**, Mbah *et al*. [66]
Moraceae	*Morus mesozygia*	Roots, stem and leaves are used to treat syphilis, dermatitis, rheumatism, asthenias, **fever and malaria**.	stem bark	90, 91 and 92	Cytotoxic and **anti-malarial**, Zelefack *et al*. [68]
	*Antiaris africana*	Bark extracts are used for the treatment of chest pain, leaf decoctions for the treatment of syphilis, and the latex is a purgative agent. It is also used in the treatment of sore throat, leprosy and **cancer**.	stem bark	93	Antioxidant and **anticancer**, Kuete *et al*. [94]
	*Treculia obovoidea*	Traditionally used to treat skin diseases, dental allergy, **amoebic dysentery** and AIDS	twigs	94	**Antimicrobial** activity, Kuete *et al*. [95]
Moraceae	*Artocarpus communis*	Treatment of cardiovascular diseases, used as food; other parts of the plants are traditionally used to treat headache, **infectious and associated diseases** such as toothache, eye problems, **ear infections**, herpes, enlarged spleen, sprains, contusions, swelling, chest pain and vomiting from heart problems, boils, abscess, and **skin infections**.	root	95 and 96	**Antimicrobial** activities, Kuete *et al*. [96]
	*Dorstenia barteri*	Used in the treatment of **malaria**.	twigs	97, 98, 99, 100, 101 and 102	**Anti-malarial** activity, Ngameni *et al*. [69]
	*Ficus cordata*	Used against hyperaesthesia, ataxia, muscle tremor, padding motions and jaundice, which could be a symptom of several related **liver diseases**.	stem bark	11, 103, 104, 105 and 106	**Hepatoprotective** and cytotoxic, Donfack *et al*. [97]
Myristicaceae	*Pycnanthus angolensis*	Treatment of stomach pain, chest pain and rhinitis problems, malaria, toothache, **fungal skin infections**, chest pain, oral thrush, and worms; some further claim a folkloric use for the treatment of leprosy.	stem bark	107, 108 and 109	**Antifungal activity**, Wabo *et al*. [98]
Olacaceae	*Coula edulis*	Treatment of stomach ache and **skin diseases**.	stem bark	110 and 111	**Antidermatophytic** activity against *Microsporum audouinii* and *Epidermophyton floccoseum*, Tamokou *et al*. [99]
Sapotaceae	*Baillonella Toxisperma*	Treatment of abscesses, infertility, stomach troubles, convulsion, rheumatism and **malaria**.	stem bark	49 and 112	Activity **against*Plasmodium falciparum***, Mbah *et al*. [70]
Verbenaceae	*Vitex cienkowskii*	Used in the treatment of many disorders, including **cardiovascular disease**.	stem bark	113	**Vasorelaxant**, antioxidant and hypertensive effect, Dongmo *et al*. [100]
Zingiberaceae	*Aframomum zambesiacum*	Used to treat **fevers**.	seeds	114, 115 and 116	**Anti-malarial**, Kenmogne *et al*. [71]
	*Reneilmia cincinnata*	Used to treat **fevers** and as a spice.	fruits	117, 118 and 119	**Anti-malarial**, Tchuendem *et al*. [72]

The isoflavonoids 3’,4’-dihydroxy-7-*O*-[(*E*)-3,7-dimethyl-2,6-octadienyl]isoflavone (75) and Griffonianone C (76), which have been isolated from *Millettia griffoniana* (used traditionally to treat female sterility, amenorrhea and menopausal disoders), both showed interesting estrogenic activities, thus validating the ethnobotanical uses of the plant [45]. Meanwhile, the anticancer properties of 3,3’-dimethoxy-4’-*O*-β-D-xylopyronosylellagic acid (93) from *Antiaris africana* (a plant traditionally used to treat cancer, amongst other ailments), has been verified. This compound indicated a pronounced tumor reducing activity (96.64%) in potato disk tumor induction assay and also showed interesting inhibitory properties against human DU-145 and hepatocarcinoma Hep G2 cells with > 70% inhibition at 50 μg/mL [94]. At the same time, the ceramide tanacetamide (113), with an interesting vasorelaxant property, was isolated along with salvin A and maslinic acid from *Vitex cienkowskii*, thus validating the use of the plant in the treatment of cardiovascular diseases [100]. The antidiabetic property of *Terminalia superba* has been verified by the isolation of 3,4’-di-*O*-methylellagic acid 3’-*O*-β-D-xylopyranoside (45) and 4’-*O*-galloy-3,3’-di-*O*-methylellagic acid 4-*O*-β-D-xylopyrano-side (46), which both exhibited good α-glucosidase inhibition [80], while the antioxidant properties of the compounds isolated from *Symphonia globulifera* could also be used to validate the use of the plant in the preparation of tonics for pregnant women [65].

Among the plants used in the treatment of diseases related to the liver, the most promising are *Ficus cordata* and *Irvingia gabonensis*, from which compounds with hepatoprotective properties have been isolated [86,97]. Among the isolated compounds, 3-friedelanone (53) might be useful for the prevention of toxin-induced and free radical-mediated liver diseases [86].

Plants used for the treatment of microbial infections have also been heavily explored. Compounds with antisalmonellal activity have been isolated from plants which have been traditionally used to treat typoid fever (an infection principally caused by *Salmonella typhi* and related baccili). The plant species include: *Turraeanthus africanus*, from which 12,15-epoxylabda-8(17),12,14-trien-16-acetate (82) has been isolated and was found to be the only active principle, possessing the minimum inhibitory concentration (MIC) and minimum bactericidal concentration (MBC) values of respectively 25 μg/mL and 100 μg/mL against *Salmonella typhi*, *S*. *paratyphi A* and *S*. *paratyphi B*[90]; and *Cassia petersiana* from which stigmasterol-3-*O*-β-D-glucoside (35) was identified as the active principle [77]. Several other plant species have been investigated for their potential for antimicrobial agents due to their uses in traditional medicine. The isolated metabolites have been tested against a wide range of Gram-positive bacteria, Gram-negative bacteria and fungi. The most convincing studies for antifungal plants were carried out by Ndom *et al*. [75], Ngounou *et al*. [76], Dzoyem *et al*. [81], Tsaffack *et al*. [83], Wabo *et al*. [98] and Tamokou *et al*. [99] on *Senecio mannii*, *Ceiba pentandra*, *Diospyros canaliculata*, *Psorospermum febrifugum*, *Pycnanthus angolensis* and *Coula edulis* respectively. Besides, the diterpenoid hardwickiic acid (74), which was isolated from *Irvingia gabonensis* (used in the treatment of gonorrhoea, among other ailments) was shown to be particularly active against *Neisseria gonorrhoeae in vitro* with an IC_50_ of 4.26 μM [85].

## Conclusions

In this paper, an attempt has been made to establish trends towards a chemotaxonomic classification of medicinal plants from Cameroon (located in tropical Africa) and to document dispersed data on plants, whose ethnobotanical uses have been validated by bioassay screening of the isolated phytochmicals. The information contained herein could serve as a starting point for further studies on Cameroonian medicinal plants. This further demonstrates that the Cameroon rainforest has a great potential for new drugs and improved plant medicines [15,19]. However, plants from a good number of families have never been investigated phytochemically. These include, among others, the Agavadeae, Amaranthaceae, Araceae, Aspidiaceae, Bamingtoniaceae, Basellaceae, Begoniaceae, Capparaceae, Caricaceae, Chenopodiaceae, Commelinaceae, Convolvulaceae, Cuburbitaceae, Ericaceae, Icacinaceae, Lecydiaceae, Loranthaceae, Lythraceae, Malvaceae, Musaceae, Nyctginaceae and Oxalicaceae, which have diverse uses in traditional medicine [20]. Moreover, a majority of the plant species studied have been harvested in the rainforests of the Southern part of the country (our statistacs show that > 95% of the isolated metabolites were derived from plants harvested in the North West, South West, Littoral and Western plateau as well as the Centre, South and East regions). However, on the basis of the fact that the Northern parts of the country represent the semi-arid and arid regions, the variations in soil composition and climatic conditions could result in dramatic variations of phytochemical compositions of the plant species and should be subject to further investigation, since the local populations in this part of the country are heavily dependent on traditional herbal medicines for their primary health care. One species which needs to be urgently subjected to phytochemical investigation is *Bridelia ferruginea*, whose leaves, barks and fruits are used for the treatment of dysentery, diabetes and as a remedy for thrush (mycotic stomatitis) in children [101,102], and as an antidote for snake bite [103]. In addition, the root decoction is also used for the treatment of gonorrhoea [104] and as an antidote for poisons [103]. The ethnobotanical use of this plant has been validated by *in vitro* activity of the leaf extracts against *Pseudomonas frutescens*, *Bacillus subtilis*, *Staphylococcus aureus*, *Streptococcus faecalis*, and *Echerichia coli*[103]. Another species that requires to be investigated is *Piper guineense*, whose antifungal properties have been verified by screening of the leaf extracts [105]. Moreover, the phytochemical contents of 26 species that are currently used for the treatment of hypertension and cardiovascular problems in the Bafia tribal people [27], could be further investigated with a view to identifying potential lead compounds for the development of drugs against cardiac problems, while those used to treat intestinal disorders in Mbalmayo, Central region [106], could also be examined for their antimicrobial potential.

## Abbreviations

ATM: African traditional medicine; HTS: highthroughput screening; R&D: Research and Development; WHO: World Health Organization; WM: Western medicine.

## Competing interests

The authors declare no conflicts of interest.

## Authors’ contributions

WS, LMM, and SMNE conceived the idea. FNK, NE, LCOO, and EM participated in the data collection. All authors contributed in the data analysis, the discussion of results and the conception of the paper. FNK wrote the first draft of the paper and all authors agreed on the final version before submission.

## Authors’ information

WS and SMNE are professors of medicinal chemistry generally interested in drug design and discovery, while SMNE also focuses organic synthesis and on natural product leads from Cameroonian medicinal plants. LMM is an associate professor of organic chemistry actively involved in the isolation and characterization of secondary metabolites from Cameroonian medicinal plants. LLL holds a PhD in environmental science and manages a Chemical and Bioactivity Information Centre (CBIC) with a focus on developing databases for information from medicinal herbs in Africa. PNJ is a retired research officer of Lhasa Ltd who currently leads the CBIC branch in Leeds, UK. FNK is a PhD student working on CADD under the joint supervision of LCOO and EM, while NE is a medical practitioner engaged in a comparative study of WM and ATM.

## Pre-publication history

The pre-publication history for this paper can be accessed here:

http://www.biomedcentral.com/1472-6882/13/147/prepub

## Supplementary Material

Additional file 1 Full list of consulted journals in constructing CamMedNP. Click here for file
