# Supplementary material for: Cameroonian medicinal plants: a bioactivity versus ethnobotanical survey and chemotaxonomic classification
Source: BMC Complement Altern Med. 2013 Jun 26;13:147. doi: 10.1186/1472-6882-13-147 (PMC3703288; doi:10.1186/1472-6882-13-147)
Supplement: Additional file 1 — Full list of consulted journals in constructing CamMedNP. [file 1472-6882-13-147-S1.doc]

# ADDITIONAL FILE

# Cameroonian medicinal plants: a bioactivity versus ethnobotanical survey and chemotaxonomic classification

### Fidele Ntie-Kang^1,2§^, Lydia L Lifongo^1^, Luc M Mbaze^3^, Nnange Ekwelle^4^, Luc C Owono Owono^5^, Eugene Megnassan^6^, Philip N Judson^7^, Wolfgang Sippl^8^, Simon M N Efange^1^.

^1^Chemical and Bioactivity Information Centre, Department of Chemistry, Faculty of Science, University of Buea, P. O. Box 63, Buea, Cameroon

^2^CEPAMOQ, Faculty of Science, University of Douala, P.O. Box 8580, Douala, Cameroon

^3^Department of Chemistry, Faculty of Science, University of Douala, P. O. Box 24157, Douala, Cameroon

^4^Klinikum Südstadt; Südring 81, 18055 Rostock, Germany.

^5^Department of Physics, Ecole Normale Supérieure, University of Yaoundé I, P.O. Box 47, Yaoundé, Cameroon

^6^Laboratory of Fundamental and Applied Physics, University of Abobo-Adjame, Abidjan 02 BP 801, Cote d’Ivoire

^7^Chemical and Bioactivity Information Centre, 22-23 Blenheim Terrace, Woodhouse Lane, Leeds, LS2 9HD, UK.

^8^Department of Pharmaceutical Sciences, Martin-Luther University of Halle-Wittemberg, Wolfgang-Langenbeck Str. 4, 06120, Halle(Saale), Germany

^§^Corresponding author

List of Journals Consulted in the Survey

| Journal type | List |
| --- | --- |
| International | *Acta Chimica Slovenica, Acta Crystallographica*, *African Journal of Biotechnology*, *African Journal of Health Sciences*, *African Health Sciences*, *Analytical Sciences*, *Annals of Tropical Medicine and Parasitology*, *Annals of Clinical Microbiology and Antimicrobials*, *Arkivoc*, *Asian Journal of Chemistry*, *Asian Journal of Traditional Medicine*, *Biochemical Systematics and Ecology*, *Bioorganic and Medicinal Chemistry*, *Bioorganic and Medicinal Chemistry Letters*, *Bioscience Biotechnology and Biochemistry*, *BMC Complementary and Alternative Medicine*, *BMC Research Notes*, *Boletín Latinoamericano y del Caribe de Plantas Medicinales y Aromáticas*, *Brazalian Journal Medical Biology Research*, *Bulletin of the Chemical Society of Ethiopia*, *Canadian Journal of Chemistry*, *Carbohydrate Research*, *Cell Division*, *Chemistry and Biodiversity*, *Chemical and Pharmaceutical Bulletin*, *Chemistry of Natural Compounds*, *European Journal of Pharmacology, European Journal of Plant Pathology*, *Fitoterapia*, *Greener Journal of Biological Sciences, Helvetica Chimica Acta*, *Indian Journal of Pharmacology*, *Inflammopharmacology*, *International Journal of Pharmacy and Pharmaceutical Sciences*, *International Journal Antimicrobial Agents*, *International Journal of Mass Spectrometry*, *Iranian Journal of Medical Sciences*, *Journal of Antibiotics*, *Journal of Brazilian Chemical Society*, *Journal of Medicinal Chemistry*, *Journal of Natural Products*, *Journal of Organic Chemistry*, *Journal of Asian Natural Products Research*, *Journal of Ethnopharmacology*, *Journal of the American Oil Chemistry Society*, *Leukemia Research*, *Malaria Journal*, *Molecules*, *Natural Product Communications*, *Natural Product Letters*, *Natural Product Research*, *Natural Product Science*, *Pakistani Journal of Medical Science*, *Parasitology Research*, *Pharmacologia*, *Pharmacologyonline*, *Pharmacopée de Médecine Traditionnelle Africaine*, *Pharmazie*, *Phytochemistry*, *Phytochemistry Letters*, *Phytochemical Analysis*, *Pharmaceutical Biology*, *Phytotherapy Research*, *Phytomedicine*, *Planta Medica*, *PLoS One*, *Pure and Applied Chemistry*, *Rasayan Journal of Chemistry*, *Records of Natural Products*, *Research Journal in Phytochemistry*, *South African Journal of Botany*, *Talanta*, *Tetrahedron*, *Tetrahedron Letters*  and *Zeitschrift für Naturforschung*. |
| Cameroonian | *Cameroonian Journal of Experimental Biology,* *Journal of the Cameroonian Academy of Sciences*, *Cameroon Journal of Biosciences* and *Les Annales des la Faculté des Sciences des l’Université de Yaoundé I.* |
